# Cromolyn platform suppresses fibrosis and inflammation, promotes microglial phagocytosis and neurite outgrowth

**DOI:** 10.1038/s41598-021-00465-6

**Published:** 2021-11-12

**Authors:** Yi-Jun Wang, Matthew A. Downey, Sungwoon Choi, Timothy M. Shoup, David R. Elmaleh

**Affiliations:** 1 AZTherapies, Inc., Boston, MA USA; 2 Department of New Drug Discovery, Chungnam National University, Daejeon, South Korea; 3 Department of Radiology, Massachusetts General Hospital and Harvard Medical School, Boston, MA 02129-2060 USA

**Keywords:** Neurogenesis, Neurodegeneration, Microglial cells, Gene regulation in immune cells, Inflammation, Neuroimmunology

## Abstract

Neurodegenerative diseases are characterized by chronic neuroinflammation and may perpetuate ongoing fibrotic reactions within the central nervous system. Unfortunately, there is no therapeutic available that treats neurodegenerative inflammation and its sequelae. Here we utilize cromolyn, a mast cell inhibitor with anti-inflammatory capabilities, and its fluorinated analogue F-cromolyn to study fibrosis-related protein regulation and secretion downstream of neuroinflammation and their ability to promote microglial phagocytosis and neurite outgrowth. In this report, RNA-seq analysis shows that administration of the pro-inflammatory cytokine TNF-α to HMC3 human microglia results in a robust upregulation of fibrosis-associated genes. Subsequent treatment with cromolyn and F-cromolyn resulted in reduced secretion of collagen XVIII, fibronectin, and tenascin-c. Additionally, we show that cromolyn and F-cromolyn reduce pro-inflammatory proteins PLP1, PELP1, HSP90, IL-2, GRO-α, Eotaxin, and VEGF-Α, while promoting secretion of anti-inflammatory IL-4 in HMC3 microglia. Furthermore, cromolyn and F-cromolyn augment neurite outgrowth in PC12 neuronal cells in concert with nerve growth factor. Treatment also differentially altered secretion of neurogenesis-related proteins TTL, PROX1, Rab35, and CSDE1 in HMC3 microglia. Finally, iPSC-derived human microglia more readily phagocytose Aβ42 with cromolyn and F-cromolyn relative to controls. We propose the cromolyn platform targets multiple proteins upstream of PI3K/Akt/mTOR, NF-κB, and GSK-3β signaling pathways to affect cytokine, chemokine, and fibrosis-related protein expression.

## Introduction

Most neurodegenerative diseases, including Alzheimer’s disease (AD), amyotrophic lateral sclerosis (ALS), multiple sclerosis (MS), and Parkinson’s disease (PD), are generally characterized by the chronic activation of the innate immune system, neuronal damage, and neuroinflammation^1^. Neuroinflammation is driven in no small part by reactive microglia, the resident macrophages of the central nervous system (CNS). When exposed to pathogens or inflammatory mediators, microglia adopt an activated phenotype and further propagate inflammation by releasing a wide range of cytokines and chemokines into the cellular environment^1,2^. Microglia polarization is inherently dynamic^3,4^, with functional outcomes mutually dependent on cross-communication between other cell types within the CNS and, by extension, their collective signals with those in the periphery^4,5^. Additionally, microglia play integral surveillance roles in the CNS, including tissue repair^5^, remodeling of ECM proteins^6,7^, and maintenance of synapses and neuronal health^8,9^.

Inflammation always precedes fibrosis. In the event of an acute injury, pathogen infiltration, or chronic inflammation in CNS tissue, fibrotic mechanisms close the wound to contain the site of injury to shield neurons from further damage and infection^10^, resulting in the formation of a glial scar. Glial scars are the primary form of fibrosis within the CNS, and microglia, astrocytes, and other local and infiltrating immune cells mediate their formation^10,11^. The inner core of the scar is composed of PDGFRβ-expressing fibroblast-like, non-neural cells believed to be sourced from differentiated pericytes from the basement membrane of the brain vasculature^12,13^. Pericytes alone and in mixed glia culture activate translocation of NF-κB p65 after exposure to LPS, IL-1β, and TNF-α^14^; a signaling pathway activated in fibroblasts associated with progression of pulmonary fibrosis^15^. As mentioned earlier, microglia may induce inflammatory activation of many cell types, including astrocytes. It was found that LPS-activated microglia secreted TNF, IL-1α, and C1q to induce A1 astrocyte activation, a neuro-inflammatory state upregulated in the aging brain directly associated with the death of axotomized neurons^16,17^. In-kind with their prominent surveillance roles in the CNS, microglia pro-inflammatory activation is well known to attract a variety of leukocytes to sites of inflammation, including T cells^9^. The severity of fibrotic scarring in an autoimmune encephalomyelitis (EAE) mouse model caused by collagen-depositing fibroblasts was regulated by T cell IFN-γ signaling^18^. Microglia readily secrete IP-10 during inflammation^19^ and also express its receptor, CXCR3, which is necessary for microglial recruitment to sites of CNS injury^20^. T-cell expression of CXCR3 and CCR5, the latter of which is a receptor for MIP-1α and MIP-1β, increases T-cell recruitment to areas of inflammation^21^. Chronic microglial activation to an inflammatory phenotype may lead to upregulation of innate CNS fibrotic mechanisms detrimental to brain parenchymal function. Therefore, modulating microglial behavior more toward an anti-inflammatory, anti-fibrotic, and phagocytic profile would be of meaningful benefit to limit neurodegeneration.

The aging brain has characteristic structural and physiological changes associated with age-related decline, including regional thickening of the basement membrane^22^ and differential changes in extracellular matrix (ECM) protein deposition^23^. Though the ECM provides critical structure to brain parenchyma to maintain stable, functional neuronal networks, studies with rat hippocampal neuron cultures suggest that reducing ECM by hyaluronidase treatment encourages mature neurons to explore and redefine network connectivity without inducing hyperexcitability even in the presence of bicuculline^24^. Early upregulation of ECM proteins in the hippocampus of APPswe transgenic AD mice was discovered to coincide with increased Aβ levels in the brain, hippocampal synapse impairment, and cognitive deficits; hippocampal injection of an ECM inactivating chondroitinase ameliorated these deficits^25^. Thus, it is possible that dynamic structuring and restructuring of the ECM in the brain that accommodates neuronal activity is dysregulated in neurodegenerative disorders^26^, and the success of surveilling the ECM for aberrant structure may rely on maintaining anti-inflammatory microglia^27,28^. Systemic inflammation modeled by daily peripheral injections of LPS in MRL/lpr mice shows that microglia migrate to the cerebral vasculature basement membrane in response to endothelial cell release of CCL5^29^. This study revealed that microglia promote BBB integrity by expressing tight junction protein CLDN5 in the early stages of inflammation but phagocytize astrocytic end-feet and promote BBB leakage in later stages of inflammation^29^. As astrocytes work in concert with microglia to contain and repair injuries in the CNS^30^, an influx of peripheral cells due to decreased BBB integrity can trigger pattern recognition receptors (PRRs) in microglia, such as toll-like receptors (TLRs) and Nod-like receptors (NLRs), to worsen inflammation^31^. Some ECM components, including fibronectin, tenascin-c, and collagen, are upregulated during fibrosis of other tissues and are ligands to PRRs of microglia recognized as damage-associated molecular patterns (DAMPs)^31–34^. Macrophages differentiated from CD14^+^ blood monocytes are a source of ECM components and ECM remodeling enzymes in the context of wound-healing in skin^35^. Several observational studies found excessive ECM remodeling and high serum levels of collagen epitopes are linked to disease progression, including collagens III and IV in idiopathic pulmonary fibrosis patients^36^ and collagens III and VI in systemic sclerosis patients^37^. Continuing the study of neurodegenerative fibrotic mechanisms may reveal early biomarkers of disease progression for preventative therapeutics. Our experiments show that HMC3 microglia are capable of robust gene expression and protein secretion of key ECM proteins, including many collagens, classes of matrix metalloproteinases (MMPs), fibronectin, and tenascin-c.

Cromolyn is a mast cell stabilizer approved for asthma treatment as a dry powder and retinal solution for eye and nasal applications. Cromolyn was recently evaluated in a phase III clinical trial for early-onset Alzheimer’s disease (AD) (NCT: NCT02547818—A Phase III Safety and Efficacy Study of ALZT-OP1 in Subjects with Evidence of Early Alzheimer’s Disease) and is currently being evaluated in a phase II clinical trial for amyotrophic lateral sclerosis (ALS) (NCT: (NCT04428775—A Phase II Safety and Biomarker Study of ALZT-OP1a in Subjects with Mild-Moderate ALS Disease). Cromolyn has been shown in vitro as an aggregation inhibitor of amyloid β-protein (Aβ) and also reduces soluble levels of Aβ in the mouse brain in vivo^38^. Additionally, cromolyn treatment to microglia promotes their migration to amyloid deposits and their subsequent anti-inflammatory phagocytosis^39^. Cromolyn is well-tolerated in healthy volunteers and can achieve CSF levels that may be sufficient to inhibit aggregation of the daily amounts of brain amyloid produced^40^. Previous studies in our group have shown that 3 μM cromolyn and F-cromolyn can reduce inflammatory cytokine and chemokine secretion by HMC3 microglia induced with TNF-α^19^. In the current study, we increased the treatment concentration to 30 μΜ to discover that IL-2, GRO-α, Eotaxin, and IL-4 are also significantly affected. We utilized unbiased proteomic profiling of secreted proteins by HMC3 microglia exposed to TNF-α and RNA-sequencing to determine the anti-fibrotic potential of cromolyn and its fluorinated analog F-cromolyn^41^ in neurodegenerative disease. Alongside cromolyn’s ability to encourage anti-inflammatory profile of microglia, this study finds that cromolyn and F-cromolyn significantly affect TNF-α-induced HMC3 microglia expression of pro-fibrotic genes, critical mediators of inflammation, amplify neurite outgrowth in PC12 neural cells in concert with nerve growth factor (NGF) and promote phagocytosis against Aβ42 of human iPSC-derived microglia. Altogether, the cromolyn platform represents a robust therapeutic strategy to address many aspects of neurodegenerative disease, particularly in multi-modal approaches required to treat multi-faceted diseases like AD and ALS.

## Methods and materials

### Chemicals and reagents

DMEM-high glucose medium (Cat#11995065), DMEM without phenol red (Cat#21063029), RPMI 1640 Medium (Cat#11875119), HS (horse serum) (Cat#26050088), NGF (nerve growth factor) 2.5S Native Mouse Protein (Cat#13257019), PBS-pH7.2 (Cat#20012027), l-glutamine (Cat#25030081), Trypsin–EDTA (Cat#25200056), and penicillin–streptomycin (Cat#15140122) were products of Gibco, Thermo Fisher Scientific. Goat anti-Rabbit IgG (H + L) Secondary Antibody-Alexa Flour 488 (Cat#A11034), Hoechst-33342 (Cat#H3570), LysoTracker Red DND-99 (Cat#L7528), and Pierce 16% Formaldehyde (Cat#28908) were products of Invitrogen, Thermo Fisher Scientific. FBS (fetal bovine serum) (Cat#F4135), BSA (bovine serum albumin) (Cat#A9647), DMSO (dimethyl sulfoxide) (Cat#D2438), Triton X-100 (Cat#T8787), and TWEEN-20 (Cat#P1379) were purchased from Sigma-Aldrich.

QIAsymphony RNA Kit (Cat#931636) and Buffer RLT Plus (Cat#1053393) were products of QIAGEN. Recombinant human TNF-α (Cat#300-01A) was purchased from PeproTech. BD Matrigel Matrix (Cat#354234) was purchased from BD Biosciences. β3-Tubulin (D71G9) Rabbit Antibody (Cat#5568) was purchased from Cell Signaling Technology. Fluorescein (FITC)-β-Amyloid-42 (Cat#A11191) was purchased from rPeptide. 3 mL Syringe/Needle Combination with Luer-Lok™ Tip (Cat#8936G82), 13 mm syringe filter (PVDF, 0.22 µm) (Cat#1159T77) were purchased from Thomas Scientific. AZTherapies provided cromolyn and F-cromolyn, and DMEM was used as the diluent to achieve final concentrations as indicated.

### Cell line and cell culture

HMC3 human microglial cell line (CRL-3304) and PC12 cell line (CRL-1721) were purchased from ATCC (American Type Culture Collection). HMC3 cells were cultured in DMEM medium with 10% FBS, 1% l-glutamine, and 1% penicillin/streptomycin (P/S) and maintained in a 37 °C incubator at 5% CO_2_. PC12 cells were cultured in RPMI-1640 medium supplemented with 10% HS, 5% FBS, and 1% P/S then placed in humidified air chamber containing 5% CO_2_ at 37 ℃. The monolayer cells were harvested by trypsin and seeded into microplates to assess the compound effect on neurite outgrowth. Human iPSC-derived microglia (Cat#BX-0900) was purchased from BrainXell.

### RNA-sequencing analysis

#### RNA isolation and sequencing

Cells collected from each condition were lysed in the Buffer RLT Plus (QIAGEN), and RNA isolation was performed on a QIAsymphony SP/AS using QIAsymphony RNA Kit according to the manufacturer instruction. Quality check and quantification were then performed on the isolated RNA samples using Agilent Fragment Analyzer. RNA samples that passed quality check were used as input for sequencing library preparation, using Illumina stranded mRNA kit per the manufacturer’s instruction. Quality check and quantification were performed on the resulting libraries, followed by sequencing on an Illumina NextSeq 550 using NextSeq High Output 75 cycles kit and single read 75 bases format. The resulting raw data were converted and demultiplexed into fastq format using Illumina bc2fastq software. FastQC analyses were performed on the demultiplexed fastq files.

#### Bioinformatics

The fastq files resulting from sequencing were used as input for downstream analysis. Briefly, reads from fastq files were mapped to the human genome (UCSC Hg38) with HISAT2 aligner, and read counting for genes was performed using featureCounts of Subread package. The results from featureCounts were used as input for differential gene expression and functional analysis in R. Differential expression analysis was performed with DESeq2. Genes differentially expressed were identified (p‐adj < 0.05 with or without a twofold differences cutoff). The list of differentially expressed genes was used as input for functional analysis, including Gene Set Enrichment Analysis comparing against Molecular Signature Database (MSigDB, BROAD Institute) and over‐representation analysis against Reactome database. Data analysis and visualization (dotplot, volcano plot, etc.) were performed with R, using DESeq2, fgsea, ReactomePA, clusterProliler, and EnhancedVolcano packages.

### Proteomics analysis using HRM-ID + mass spectrometry

#### Sample preparation

Proteins from 800 µl of each sample were precipitated with acetone overnight at − 20 °C. Proteins were then denatured using Denature Buffer (Biognosys) and reduced and alkylated using Reduction and Alkylation Solution (Biognosys) for 60 min at 37 °C. Subsequently, digestion to peptides was carried out using trypsin (Promega, 1:50 protease to total protein ratio) overnight at 37 °C.

#### Clean-up for mass spectrometry

Peptides were desalted using BioPureSPN C18 MINI spin columns (The Nest Group) according to the manufacturer’s instructions and dried down using a SpeedVac system. Peptides were resuspended in LC solvent A (1% acetonitrile, 0.1% formic acid (FA)) and spiked with iRT kit calibration peptides (Biognosys). Peptide concentrations were determined using a UV/Vis Spectrometer at 280 nm (SPECTROstar Nano, BMG Labtech).

#### HRM (DIA) mass spectrometry acquisition

For DIA LC–MS/MS measurements, 1 µg of peptides per sample was injected into an in-house packed, reversed-phase column (PicoFrit emitter with 75 µm inner diameter, 60 cm length and 10 µm tip from New Objective, packed with 1.7 µm Charged Surface Hybrid C18 particles from Waters) on a Thermo Scientific™ EASY-nLC™ 1200 nano-liquid chromatography system connected to a Thermo Scientific™ Orbitrap Fusion™ Lumos™ Tribrid™ mass spectrometer equipped with a Nanospray Flex™ Ion Source. LC solvents were A: 1% acetonitrile in water with 0.1% FA; B: 20% water in acetonitrile with 0.1% FA. The nonlinear LC gradient was 1–59% solvent B in 95 min followed by 59–90% B in 10 s, 90% B for 8 min, 90–1% B in 10 s, and 1% B for 5 min at 60 °C and a flow rate of 250 nL/min. The DIA method consisted of one full-range MS1 scan, and 29 DIA segments were adopted from Bruderer et al.^42^.

#### HRM data analysis

HRM data were first analyzed using a directDIA search using Biognosys’ search engine SpectroMine, the false discovery rate on peptide and protein level was set to 1%. A human UniProt .fasta database (Homo sapiens, 2020-07-01) was used for the search engine, allowing for two missed cleavages and variable modifications (N-term acetylation, methionine oxidation). HRM mass spectrometric data were analyzed using Spectronaut™ 14 software (Biognosys). The false discovery rate on peptide and protein level was set to 1%, and data was filtered using row-based extraction. The assay library (protein inventory) generated in this project was used for the analysis. The HRM measurements analyzed with Spectronaut were normalized using local regression normalization^43^; separate normalization was performed for three control samples.

For testing of differential protein abundance, protein intensities for each protein were analyzed using a two-sample sample Student’s t-test. The following thresholds were applied for candidate identification: p-value < 0.05; absolute average log2 ratio > 0.58 (fold-change > 1.5). Principal component analysis was conducted in R using *prcomp* and a modified *ggbiplot* function for plotting, and partial least squares discriminant analysis (PLS-DA) was performed using the *mixOMICS* package. PLS-DA model fitting was performed on the samples from stimulated cells. Functional analysis was performed using String-db (string-db.org, version 11). General plotting was done in R using the *ggplot2* package.

### Neurite outgrowth assay

96 well plate was coated with 50 μL/well 1% Matrigel in DMEM at 4 ℃ overnight. PC12 cells were plated into the coated plate at a density of 3 × 10^3^ cells/well in a 100 μL growth medium and cultured for 24 h. Cells were incubated with 200 μL/well of fresh medium containing test compounds for 7 days followed by fresh medium replacing every 3 days. Cells were fixed with 4% paraformaldehyde and incubated for 20 min at room temperature (RT), then they were rinsed in PBS and incubated with permeabilization buffer (0.1% Triton-X 100 in PBS) for 20 min at RT. Followed PBS washing, cells were incubated with β3-Tubulin primary antibody (1:1000) in antibody buffer (3% BSA in PBS + 0.05% Tween) at 4 ℃ overnight. Cells were rinsed with PBST (PBS + 0.05% Tween) and incubated with secondary antibody (1:1000) and Hoechst (1:1000) in antibody buffer for 2 h at RT away from light. After PBS washing, cells images were taken by the High-Content Imaging System, CellInsight CX5 (ThermoFisher Scientific). The nuclei were stained by Hoechst, and the neuron cell bodies and neurites were stained with secondary antibodies conjugated Alexa Fluor 488. 10× objective was used, and 9 fields/well were captured in both nuclei and neurite channels. The neurite analysis module of HCS measured the average cell number and neurite length per field.

### Human iPSC-derived microglial phagocytosis assay of amyloid β-42

Human iPSC-induced microglia (BrainXell, BX-0900) were cultured in microglia culture medium for 5 days according to the manufacturer’s instruction. FITC-labeled Aβ42 proteins were dissolved with 2 mM NaOH and diluted with PBS to 30 μM followed by aging in the dark at 37 °C for 2 h to form aggregated FITC-Aβ42. The microglia were treated with compounds at different concentrations for 30 min, followed by the addition of aggregated FITC-Aβ42 to a final concentration of 0.3 μM. After incubation for 24 h, the microglia were fixed with 4% PFA at RT for 20 min and then subjected to staining solution containing 1 μg/mL Hoechst-33342 and 100 nM LysoTracker™Red DND 99 in PBS at RT for 5 min. The fluorescent images were acquired under 10× objective using PE Operetta CLS System, and the positive cell percentage with phagocytosed FITC-Aβ42 was quantified.

### Detection of secreted proteins using the MSD assay platform

HMC3 cells were resuspended, counted using the LUNA-II Automated Cell Counter, seeded in the 6-well plate (400 K cells/2 mL medium/well), and incubated overnight to allow cells to attach. The media and detached cells were removed the next day. The cell layer was washed twice in PBS and once in serum- and phenolred-free DMEM (SPFM). Cells were incubated in SPFM for 4 h prior to treatment with TNF-α (0.3 µg/ml) and/or Cromolyn (0.3 µM, 3 µM, 10 µM, 30 µM) or F-cromolyn-diacid (0.3 µM, 3 µM, 10 µM, 30 µM) for 24 h. The conditioned medium was collected and centrifuged at 1000× RCF (*g*) for 5 min to pellet detached cells and large debris, which was subsequently passed through a 0.22 µm filter with PVDF membrane to remove smaller debris. Samples of the supernatant medium were put in a CoolRack (#07210041, Fisher Scientific) on dry ice for Snap-freezing and kept at − 80 °C until use.

Meso-scale V-PLEX plates that detect a panel of human cytokines including IL-2, IL-4, VEGF-A, and a panel of human chemokines including GRO-α/CXCL1, Eotaxin/CCL11, were used as per the manufacturer’s protocol. 25 µL of the conditioned medium was used in each well of the MSD plates to detect the analytes. The plates were then analyzed on an MSD QuickPlex SQ120 instrument.

### PI3K-δ lipid kinase activity via ADP-Glo enzyme assay

Activity-based PI3K-δ (p110d/p85a) lipid kinase assay kit for compound testing via ADP-Glo kinase system was used according to the manufacture’s protocol (Reaction Biology). The PI3K-δ enzyme was diluted in prepared PI3K-δ Reaction Buffer/Lipid Substrate mixture. 0.5 μL of testing compound solution, 4 μL of enzyme/lipid mixture, and 0.5 μL of 250 μM ATP in water were added to the wells of a 384-well low volume plate and incubated at RT for 60 min. 5 μL of ADP-Glo Reagent with 10 mM MgCl_2_ were added to each well and incubated at RT for 40 min, followed by addition of 10 μL Kinase Detection Reagent and incubation at RT for another 30 min. Luminescence was recorded as the readout for kinase activity.

### Quantification and statistical analysis

All the data were presented as mean ± standard error from at least three times, each done in triplicate. The statistical significance between two groups was determined by Student’s t test, whereas the comparisons of multiple groups were carried out by one-way ANOVA, followed by Bonferroni’s post-test using GraphPad Prism 7 (GraphPad Software, Inc.). A probability value of *p < 0.05 was considered to be significant.

## Results

### TNF-α is a robust inducer of differentially expressed genes and proteins in HMC3 human microglia

**Figure 1:**
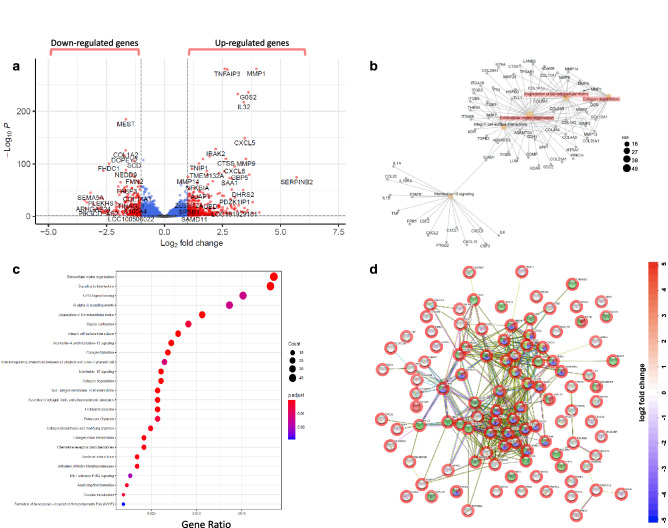
TNF-α potently induces differential expression of a wide array of fibrosis and inflammation-associated genes and proteins. (**a**) The volcano plot includes 26,485 genes quantified, with red dots showing gene candidates. 1061 genes are differentially expressed upon addition of 0.3 µg/mL TNF-α to HMC3 microglia. Cutoff is log_2_ fold change (> 2 folds) and p-value < 0.05. (**b**) Function-to-Gene association Network (FGNet) plot presents genes significantly altered by TNF-α as they relate to the indicated cellular function. (**c**) Pathway enrichment dotplot collects pathway associations among significantly altered genes after TNF-α administration. The dot size represents the number of genes related to the indicated pathway whose expressions were significantly changed, and its color indicates its statistical significance. (**d**) String-db functional analysis of Protein–Protein Interaction Network (PPINet). Each spherical node is a protein significantly changed after TNF-α administration to HMC3 cells, with the color aura of the sphere indicating log_2_ fold change from HMC3 cells alone. Each sphere is annotated by color: red for response to cytokines, blue for cytokine-mediated signaling pathway, green for response to stress, and yellow for chemokine-mediated signaling pathway. Each edge represents a PPI, whether experimentally determined or known from curated databases, or the edge represents predicted protein interactions based on genomic or proteomic neighborhood, fusions, or co-occurrence.

RNA-seq analysis found the response by HMC3 microglia to 0.3 µg/mL TNF-α resulted in differential expression of 1061 genes out of 26,485 genes quantified (Fig. 1a). Interestingly, Function-to-Gene Association Network (FGNet) analysis revealed TNF-α affects many cellular functions, including organization and degradation of the extracellular matrix (ECM), which have been associated with fibrotic diseases^44^, as well as collagen degradation (Fig. 1b). Many genes were differentially altered, including MMP1, MMP3, MMP9, CXCL2, CXCL5, CXCL8, CXCL10, IL-6, COL21A1, COL12A1, COL1A2, and TGFB2 (Fig. 1b and Supplementary Figure S1a). This observation is further highlighted by a pathway enrichment dotplot in Fig. 1c, showing that 40 or more genes significantly changed by TNF-α are involved in ECM organization and interleukin signaling, respectively; 20 or more genes each associated with degradation or formation of collagen and collagen fibril assembly, respectively; and 20 or more genes involved with ECM proteoglycans. The extent of interconnected cellular networks of fibrosis affected by TNF-α treatment of HMC3 microglia is shown in Supplementary Figure S1b, with an enriched pathway network plot showing interconnections of ECM organization, collagen biosynthesis/assembly/formation, and ECM degradation. Unbiased quantification of all detectable peptides and proteins via HRM-ID + mass spectrometry resulted in 3730 proteins, 42,435 peptides, and 59,076 peptide ion variants across all samples tested. Out of these, a total of 3427 proteins were changed significantly. String-db functional analysis of Protein–Protein Interaction Network (PPINet) incorporating known and predicted interactions shows a complex, dense network of connections between significantly-altered proteins in HMC3 microglia due to TNF-α (Fig. 1d and Supplementary Figure S1c). There is an extensive number of edges that interconnect between many proteins related to stress response (green sphere), cytokine signaling (blue spheres), response to cytokines (red spheres), and chemokine signaling (yellow spheres). The dense network of strings in the PPINet analysis indicates that TNF-α elicits a robust response in HMC3 microglia that likely affects more biologically connected proteins than a random assortment of proteins would^45^.

### Cromolyn and F-cromolyn suppress TNF-α-induced fibrosis and inflammation in HMC3 microglia

**Figure 2:**
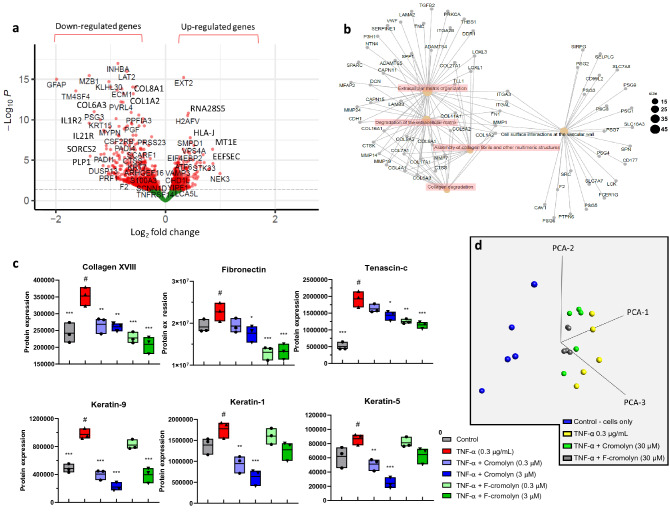
Cromolyn reduces the expression of fibrosis and inflammation-associated genes and proteins induced by TNF-α in HMC3 human microglia. (**a**) Volcano plot showing 30 µM cromolyn addition to 0.3 µg/mL TNF-α induced HMC3 microglia differentially changed expression of 1441 genes with cutoff p-value < 0.05. (**b**) FGNet analysis shows that 30 µM cromolyn addition to TNF-α treated HMC3 microglia affects the indicated cellular function. (**c**) Cromolyn and F-cromolyn significantly reduce fibrosis-associated protein secretion by TNF-α-induced HMC3 microglia, including collagen XVIII, fibronectin, and tenascin-c, and keratin-9, -1, and -5. Quantitative analysis of secreted proteins relative to the # group of TNF-α alone. One-way ANOVA was used for statistics: ***p < 0.001, **p < 0.01, *p < 0.05 versus the # group. (**d**) 3D principal component analysis (PCA) shows that cromolyn and F-cromolyn significantly alter TNF-α-induced collective gene expression in HMC3 microglia compared to the cells alone. The plotted points are condensed representations of gene expression, with the 0.3 µg/mL TNF-α treatment cluster (yellow) most spatially distant from the control cluster (blue). 30 µM Cromolyn (green) and 30 µM F-cromolyn (grey) groups are clustered together.

As TNF-α was found to exhibit a pro-fibrotic and pro-inflammatory profile for HMC3 microglia in our experiments, we next sought to determine cromolyn’s ability to influence this process. The RNA-sequencing analysis found that adding 30 µM cromolyn to 0.3 µg/mL TNF-α-induced HMC3 microglia differentially regulated the expression of 1441 genes with a cutoff p-value < 0.05 (Fig. 2a). Furthermore, FGNet analysis revealed that 30 µM cromolyn significantly affects cellular pathways associated with ECM organization, degradation of ECM, assembly of collagen fibrils, and collagen degradation (Fig. 2b; Supplementary Figures S2a,b).

As a majority of the genes altered by cromolyn affect ECM dynamics, we next performed proteomics analyses using HRM-ID + mass spectrometry of the cell supernatant of TNF-α-treated HMC3 microglial cells in the presence of cromolyn or F-cromolyn (Fig. 2c). We found that TNF-α induced secretion of collagen XVIII alpha-1 (COL18A1) relative to control (p = 0.0009) and 0.3 µM and 3 µM cromolyn treatment achieved similar decreases in COL18A1 secretion (p = 0.0094 and p = 0.005, respectively) (Fig. 2c). 0.3 µM F-cromolyn significantly decreases COL18A1 further (p = 0.0003) than either concentration of cromolyn relative to TNF-α treatment alone and 3 µM F-cromolyn decreases COL18A1 secretion further still (p < 0.0001) (Fig. 2c). We also found that addition of 0.3 µM and 3 µM cromolyn after TNF-α treatment to HMC3 microglia mildly reduced fibronectin secretion, but the reduction of fibronectin caused by 0.3 µM and 3 µM F-cromolyn addition was more significantly pronounced (p = 0.0004 and p = 0.0005, respectively) (Fig. 2c). Additionally, we observed that ECM component Tenascin-c (TNC) secretion was strongly upregulated by 0.3 µg/mL TNF-α (p < 0.0001) (Fig. 2c). Though addition of 0.3 µM or 3 µM cromolyn modestly reduced TNC secretion, 0.3 µM and 3 µM F-cromolyn significantly reduced TNC secretion by TNF-α induced HMC3 microglia (p = 0.0011 and p = 0.0003, respectively) (Fig. 2c). Interestingly, only cromolyn (0.3 µM and 3 µM) significantly reduced secretion of the keratin proteins KRT9 (p < 0.0001 and p < 0.0001), KRT1 (p = 0.0034 and p = 0.0002) and KRT5 (p = 0.0095 and p < 0.0001) post TNF-α treatment (Fig. 2c). Figure 2d shows a 3D principal component analysis (PCA) of the collective gene expression of HMC3 microglia controls relative to all treatment groups. More separation between clusters of points represents more dissimilar gene expression profiles. 30 µM cromolyn (green cluster) and 30 µM F-cromolyn (grey cluster) groups separate from the TNF-α group (yellow cluster) in the PCA plot from the control group (blue cluster). These findings highlight that across five independent experiments, cromolyn and F-cromolyn differentially alter the collective gene expression of TNF-α induced HMC3 microglial cells.

**Figure 3:**
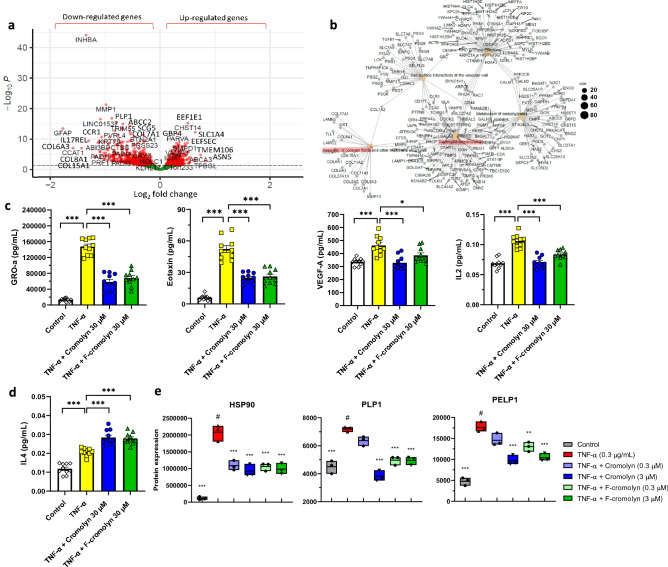
F-cromolyn reduces the expression of fibrosis- and inflammation-associated genes and proteins induced by TNF-α in HMC3 human microglia. (**a**) Volcano plot of 2108 genes differentially altered by 30 µM F-cromolyn after 0.3 µg/mL TNF-α addition. (**b**) FGNet analysis of cellular pathways significantly affected by F-cromolyn. Dot size is indicative of the approximate number of genes grouped with a particular cellular pathway. (**c**) Cytokine secretion of HMC3 microglia controls (white diamonds), after addition of 0.3 µg/mL TNF-α (yellow squares), 0.3 µg/mL TNF-α + 30 μM cromolyn (blue circles), and 0.3 µg/mL TNF-α + 30 μM F-cromolyn (green triangles) for GRO-α, Eotaxin, VEGF-A, IL-2, and (**d**) IL-4. ***p < 0.001, **p < 0.01, *p < 0.05. (**e**) Cromolyn and F-cromolyn significantly reduce pro-inflammatory protein secretion by TNF-α-induced HMC3 microglia, including HSP90, PLP1, and PELP1. Quantitative analysis of secreted proteins relative to # group of TNF-α alone. One-way ANOVA was used for statistics: ***p < 0.001, **p < 0.01, *p < 0.05 versus the # group.

Next, we determined through RNA-seq analysis that 30 µM F-cromolyn administration to 0.3 µg/mL TNF-α-induced HMC3 microglia differentially expressed 2108 genes using a cutoff p-value < 0.05 (Fig. 3a). Performing an FGNet analysis confirmed that 30 µM F-cromolyn affects fibrosis and inflammation-related cellular pathways, including collagen fibril assembly and neutrophil degranulation (Fig. 3b; Supplementary Figure S3a,b). MSD V-Plex cytokine assays for GRO-α, Eotaxin, VEGF-Α, IL-2, and IL-4 in the presence of 30 µM cromolyn and F-cromolyn are provided in Fig. 3c,d. TNF-α potently induced GRO-α secretion in HMC3 microglia (p < 0.0001). 30 µM cromolyn and 30 µM F-cromolyn each significantly reversed GRO-α secretion induced by TNF-α treatment (p < 0.0001 and p < 0.0001, respectively) (Fig. 3c and Supplementary Figure S3c). TNF-α treatment also significantly increased Eotaxin expression (p < 0.0001) and administration of either 30 μM cromolyn or 30 μM F-cromolyn decreased Eotaxin levels by approximately 40% (p < 0.0001 and p < 0.0001, respectively) (Fig. 3c). We also observe that 0.3 µg/mL TNF-α treatment increased secretion of VEGF-Α in HMC3 microglia (p < 0.0001), but 30 µM cromolyn reduced VEGF-A secretion to pre-TNFα treatment levels (p < 0.0001). Additionally, 30 µM F-cromolyn reduced VEGF-A secretion after TNF-α treatment (p < 0.0186) (Fig. 3c). Although in a previous study^19^ we did not observe significant upregulation of IL-2 or IL-4 in the presence of 0.3 µg/mL TNF-α, we were able to show over many repeat experiments that TNF-α can significantly increase secretion of both cytokines (Fig. 3c,d). IL-2 secretion by HMC3 microglia is promoted following 0.3 µg/mL TNF-α administration (p < 0.0001), but is significantly decreased following 30 µM cromolyn or 30 µM F-cromolyn treatment (p < 0.0001 and p < 0.0001, respectively) (Fig. 3c). 30 µM cromolyn and F-cromolyn significantly increased IL-4 secretion after 0.3 µg/mL TNF-α treatment (p < 0.0001 and p < 0.0001, respectively) (Fig. 3d). This finding is important as IL-2 is a pro-inflammatory cytokine with prominent roles in T-cell proliferation and differentiation^46^ and IL-4 is an anti-inflammatory cytokine capable of suppressing NF-κB signaling in monocytes to inhibit secretion of pro-inflammatory cytokines IL-6 and IL-1β^47^.

HRM-ID + mass spectrometric proteomic assays determined that TNF-α increases PLP1 secretion by HMC3 microglia (Fig. 3e). Although 0.3 µM cromolyn did not significantly change PLP1 secretion, 3 µM cromolyn drastically decreases average PLP1 secretion below control levels (p < 0.0001). Both 0.3 µM and 3 µM F-cromolyn also decrease PLP1 secretion relative to TNF-α treatment alone (p < 0.0001 and p < 0.0001), though there is no apparent difference in inhibition based on concentration. We also observed increased secretion of PELP1 after 0.3 µg/mL TNF-α treatment. 0.3 µM cromolyn modestly decreases PELP1 secretion induced by TNF-α, but increasing concentration to 3 µM cromolyn significantly reduces PELP1 levels (p < 0.0001). Repeating the experiment with 0.3 µM and 3 µM F-cromolyn achieved similar decreases in PELP1 secretion (p = 0.0049 and p < 0.0001) as to what was observed with respective concentrations of cromolyn (Fig. 3e). We also found that TNF-α administration strongly induces HSP90 secretion in HMC3 microglia (p < 0.0001). Subsequent treatment with either 0.3 µM or 3 µM concentrations of cromolyn or F-cromolyn resulted in a significant decrease in HSP90 secretion after TNF-α administration (p < 0.0001, respectively).

### Cromolyn and F-cromolyn augment NGF-induced neurite outgrowth in PC12 neuronal cells

**Figure 4:**
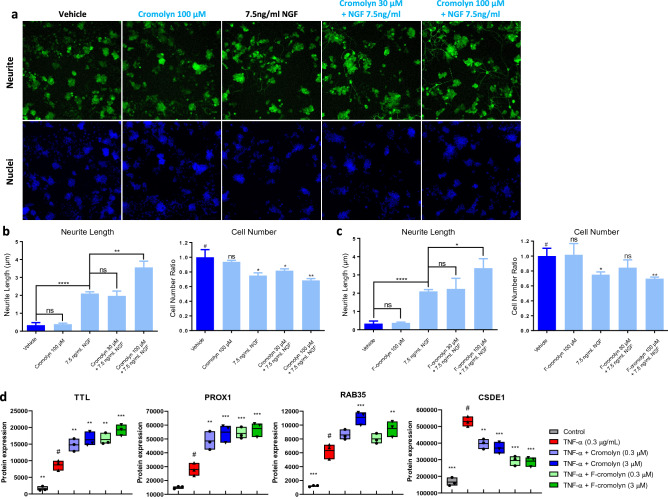
Cromolyn and F-cromolyn promote neurite outgrowth and neurogenesis in PC12 cells and differentially alter neurogenesis-related proteins in HMC3 microglia. (**a**) Top row incorporates PC12 cell staining with Alexa Fluor 488 for β3-tubulin (green) for imaging of neurite outgrowth over vehicle, 100 µM cromolyn, 7.5 ng/mL nerve growth factor (NGF), 30 µM cromolyn + 7.5 ng/mL NGF, and 100 µM cromolyn + 7.5 ng/mL NGF conditions. The bottom row incorporates Hoechst staining for nuclei of PC12 cells for comparison over the same treatment conditions. (**b**, **c**) Neurite length bar plots and cell number measurements normalized to vehicle for PC12 cells over the indicated treatment condition, including (**b**) cromolyn and (**c**) F-cromolyn. ****p < 0.0001, **p < 0.01, *p < 0.05, ns = no significance. (**d**) Cromolyn and F-cromolyn significantly regulate the expression of neurite outgrowth and neurogenesis-associated proteins by TNF-α-induced HMC3 microglia, including TTL, PROX1, RAB35, and CSDE1. Quantitative analysis of secreted proteins relative to # group of TNF-α alone. One-way ANOVA was used for statistics: ***p < 0.001, **p < 0.01, *p < 0.05 versus the # group.

As the reduction in neuron processes is characteristic of neurodegeneration^48^, we sought to determine the effect of cromolyn and F-cromolyn on the neurite outgrowth of PC12 neuronal cells. Figure 4a shows PC12 cells maintain similar morphology when exposed to 100 µM cromolyn compared to vehicle, suggesting that cromolyn alone does not have a measurable effect on neurite outgrowth. As expected, treating PC12 cells with 7.5 ng/mL nerve growth factor (NGF) (see Supplementary Figure S4 for NGF concentration optimization) results in noticeable extension of neurites (p < 0.0001) (Fig. 4b). Although concomitant addition of 30 µM cromolyn with NGF does not appear to change PC12 cell morphology, increasing the concentration of cromolyn to 100 µM results in a significant extension of neurite length (p = 0.0032) (Fig. 4b). We also observed decreases in cell numbers with the treatment of NGF (Fig. 4b), which is known to be due to the anti-proliferative signaling of NGF^49^ and not due to any cromolyn cytotoxicity. Under NGF treatment, PC12 cells differentiated into neuron-like cells that cannot proliferate, so the cell number of NGF addition groups was less than the vehicle group. We observe similar results in neurite outgrowth for identical experiments with F-cromolyn (Fig. 4c). Altogether, these data indicate that higher concentrations of cromolyn and F-cromolyn augment the capability of NGF to promote neurite outgrowth in PC12 neuronal cells.

HRM-ID + mass spectrometric proteomic assays determined that TNF-α increases tubulin tyrosine ligase (TTL) secretion by HMC3 microglia (p = 0.0024) (Fig. 4d). Addition of 0.3 µM and 3 µM cromolyn significantly increased TTL secretion after 0.3 µg/mL TNF-α exposure (p = 0.0088 and p = 0.0015, respectively). F-cromolyn is more effective at increasing TTL secretion than cromolyn is, as 0.3 µM F-cromolyn (p = 0.0014) achieves similar TTL levels to that of 3 µM cromolyn. Increasing to 3 µM F-cromolyn continues this trend and results in the highest TTL level observed in our experiments, relative to TNF-α administration alone (p < 0.0001). We also observed increased PROX1 secretion after addition of TNF-α (p = 0.0784) and significantly higher PROX1 levels in concert with 0.3 µM cromolyn (p = 0.0033) (Fig. 4d). 3 µM cromolyn slightly decreased PROX1 levels relative to 0.3 µM cromolyn, but still achieved significantly higher secretion of PROX1 relative to TNF-α alone (p = 0.0003). Both 0.3 µM and 3 µM F-cromolyn achieved similar increases in PROX1 secretion relative to TNF-α alone (p = 0.0005 and p = 0.0002, respectively). Further, we observed significant increase in Rab35 secretion after administering TNF-α to HMC3 microglia (p = 0.0001) (Fig. 4d). Although only a slight increase in secretion was noted for 0.3 µM cromolyn, 3 µM cromolyn increased Rab35 secretion significantly relative to TNF-α alone (p = 0.0002). 0.3 µM F-cromolyn treatment resulted in modest increase of Rab35 secretion relative to TNF-α alone, but the higher 3 µM F-cromolyn treatment had significant increase (p = 0.0074). Lastly, we noted a strong increase in CSDE1 secretion upon 0.3 µg/mL TNF-α treatment (p < 0.0001) (Fig. 4d). Addition of 0.3 µM or 3 µM cromolyn achieved similar decreases in CSDE1 relative to TNF-α alone (p = 0.0016 and p = 0.0003). Interestingly, F-cromolyn treatment at both 0.3 µM and 3 µM achieved similar significant decreases in CSDE1 relative to TNF-α (p < 0.0001 and p < 0.0001), but both resulted in lower CSDE1 secretion levels than either concentration of cromolyn.

### Cromolyn and F-cromolyn promote iPSC-derived human microglial phagocytosis of Amyloid β-protein 42 (Aβ42) via PI3K signaling

**Figure 5:**
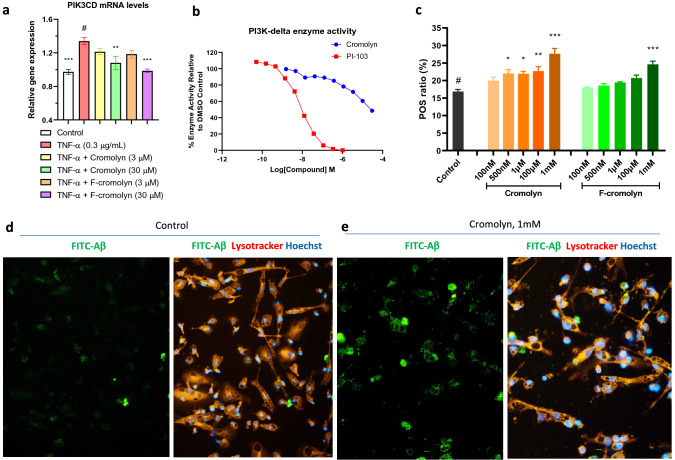
Cromolyn and F-cromolyn promote iPSC-derived human microglial phagocytosis of amyloid β-protein 42 (Aβ42) via PI3K signaling. (**a**) RNA-seq analysis shows that cromolyn and F-cromolyn decreased the gene expression levels of PIK3CD induced by TNF-α in HMC3 human microglia. Fold change is normalized to untreated control. GAPDH was used as an internal control to normalize the mRNA level of the gene being measured in each sample. Quantitative analysis of PIK3CD gene expression relative to # group of TNF-α alone. One-way ANOVA was used for statistics: *p < 0.05, **p < 0.01, ***p < 0.001 versus to # group. (**b**) Cromolyn inhibited the activity of PI3K-δ lipid kinase in a concentration-dependent manner. Compound PI-103 is used as a positive control inhibitor of PI3K-δ. (**c**) Comparison of microglia internalization of FITC-Aβ at indicated concentrations for both cromolyn and F-cromolyn. POS ratio = (cell number with phagocytosed Aβ42)/(total cell number). *p < 0.05, **p < 0.01, ***p < 0.001. (**d**) (left) Control image with fluorescein-labeled Aβ42 (FITC-Aβ) with human microglia and (right) an overlay image with FITC-Aβ (green), lysotracker for cell lysosome (orange), and Hoechst nuclei stain (blue). (**e**) 1 mM cromolyn treatment to microglia. (left) image of FITC-Aβ only and (right) overlay image with FITC-Aβ (green), lysotracker for cell lysosome (orange), and Hoechst nuclei stain (blue).

Microglia are the resident macrophages of the CNS, and the promotion of their phagocytic profile is important to limit the extent of inflammatory activation that can lead to neurodegeneration. This is particularly important as aggregate extracellular amyloid β-protein is largely associated with neuron toxicity. PI3K signaling is regulated by histone modifications^50^, and deletion of histone deacetylases 1 and 2 have led to increased microglial phagocytosis of amyloid plaques in FAD DKO mice models^51^. We found through RNA-seq analysis that both 30 μM cromolyn and 30 μM F-cromolyn significantly decreased PIK3CD mRNA levels in HMC3 microglia (p = 0.0065 and p = 0.0002, respectively) (Fig. 5a). In light of this finding, we confirmed via PI3K-δ lipid kinase assay that cromolyn dose-dependently reduced PI3K-δ enzyme activity (Fig. 5b). We then utilized iPSC-derived human microglia to ascertain the ability of cromolyn and F-cromolyn to promote microglial phagocytosis. Firstly, we determined the microglial purity of these iPSC-differentiated microglial cells to be more than 95% using IF staining for IBA-1, which is considered a marker of microglia (Supplementary Figure S5a). Secondly, we determined to use the microglial cell number of 2000 cells/well and the FITC-labeled Aβ42 concentration of 0.3 µM to conduct compound evaluation (Supplementary Figure S5b). In determining the concentration to use for the promotion by cromolyn and F-cromolyn to promote microglia phagocytosis, we compared increasing concentrations of each compound to the quantitative phagocytosis (POS) ratio; higher POS ratio corresponds to more Aβ internalization compared to all cells in the image (Fig. 5c). We found that 1 mM treatment for both cromolyn and F-cromolyn resulted in the most significant change in POS ratio relative to 0.2% DMSO control (p < 0.0001 and p = 0.0001, respectively); thus, we used 1 mM concentration to show cromolyn and F-cromolyn’s modulatory effects on microglial phagocytosis. The left panel of Fig. 5d shows the FITC-Aβ42 image of control iPSC microglia. FITC-Aβ is distributed fairly evenly throughout the image, with few spots of increased localization observed. Comparing the left panel of Fig. 5d to the right image overlay of Hoechst stain for nuclei (blue) and lysotracker for cell lysosomes (orange), it becomes apparent that clumping of FITC-Aβ resides within the lysosomes of the microglia. Upon adding 1 mM cromolyn to iPSC-derived microglia, the fluorescence of FITC-Aβ is brighter and more concentrated in specific areas of the image (Fig. 5e, left). Comparing to the right overlay image (Fig. 5e, right), it is clear that microglia have upregulated their phagocytosis of FITC-Aβ by the intense colocalization of Aβ (green) within the lysosome (orange). Because the change in POS ratio for 1 mM cromolyn and 1 mM F-cromolyn are very similar, we only show representative images for 1 mM cromolyn.

## Discussion

Attenuating inflammatory signaling is a preemptive strategy to avoid downstream neurodegenerative processes such as inflammation-induced neuronal damage and fibrosis. One such route is the PI3K/Akt/mTOR inflammatory signaling pathway^46^. We have previously shown that TNF-α treatment to HMC3 microglia induces a pro-inflammatory phenotype with significantly increased expression of IBA1 and GFAP and increased mRNA levels of CD11b and TMEM119^19^. Additionally, cromolyn and F-cromolyn significantly reduces the secretion of an array of important cytokines and chemokines, including IL-1β, IL-6, IL-8, IFN-γ, CCL2, CCL3, CCL4, and CXCL10^19^. IL-2 is another pro-inflammatory cytokine that can activate NF-κB to promote the expression of IL-6 and MCP-1 and disrupt the integrity of the blood–brain barrier by comprising components of the adherens junctions^52^. In contrast, IL-4 has been associated with protective mechanisms of brain ischemia and can induce anti-inflammatory M2 microglial phenotype expression in primary rat cortical glia cultures and encourage PPARγ-dependent microglial phagocytosis^53^. Increased expression of GRO-α (also known as CXCL1) in monocytes from APP/PS1 transgenic mice and AD patients has been implicated in increased monocyte infiltration into the brain^54^ and massive neutrophil recruitment in other models of brain and tissue damage^55,56^. The inflammatory cytokine Eotaxin is found in many cell types, including microglia and astrocytes, with roles in neurogenesis suppression and is expressed in heightened levels in aged human plasma and CSF^57^. Additionally, increased levels of VEGF have also been found in CSF and serum of AD patients relative to controls^58,59^. LPS-activated microglia from SD rats have been shown to upregulate VEGF-A expression, increase angiogenesis and promote the migration and proliferation of retinal microvascular endothelial cells while impairing tight junction protein expression^60^. In the context of prolonged inflammation, weakening of the BBB and increasing proliferating cells into the brain that respond to inflammatory stimuli could allow for increased microglial inflammatory activation. Though our previous study^19^ showed cromolyn and F-cromolyn had no significant change in IL-2, GRO-α, and Eotaxin levels at 3 μM concentration, we found increasing the concentration to 30 μM resulted in a significant reduction of all three of these cytokines. This report shows that 30 μM cromolyn and F-cromolyn suppress TNF-α-induced secretion of the three pro-inflammatory mediators IL-2, GRO-α, Eotaxin, and VEGF-A, and increase the expression of anti-inflammatory IL-4. This finding indicates that cromolyn and F-cromolyn can modulate key mediators of neuroinflammation in HMC3 microglia related to downstream neurodegenerative disease progression.

Unbiased proteomic profiling of HMC3 microglia found that cromolyn and F-cromolyn also modulate other inflammatory proteins. Proteolipid protein 1 (PLP1) is the major form of myelin protein in the CNS with roles in myelin sheath structure, oligodendrocyte proliferation, and axonal survival^61^. Plp1tg mouse models exhibited heightened levels of PLP1 and significant microglial inflammation activation throughout brain parenchyma before myelinated fibers were present, leading to 24-fold TNF-α and sevenfold IL-6 increases compared to wildtype mice^62^. Therefore, decreases in PLP1 gene expression due to cromolyn (− 14.62 fold change decrease after TNF-α exposure, Supplementary Figure S2a) may be responsible, in part, for its anti-inflammatory capabilities. We also found that cromolyn and F-cromolyn decrease Proline, Glutamate and Leucine Rich Protein 1 (PELP1) secretion following TNF-α administration. PELP1 is widely expressed in the brain, particularly in the cell membrane, dendritic shafts, and synaptic terminals of neurons in the forebrain^63^. PELP1 has been associated with pro-survival mechanisms after ischemic injury by activating ERK and Akt pathways, as well as E2 inhibition of GSK3β, a key mediator of cell death in AD^63^. In addition, PELP1 can promote NF-κB gene expression to activate macrophages^64^. PELP1 knockdown in medulloblastoma cells results in downregulation of NF-κB, including TRAF1 and IL-8, and expression of ECM-related genes MMP7, MMP9, and MMP14^65^, the latter of which is upregulated by microglia in late-stage NDD development^66^. Interestingly, PELP1 is a substrate of GSK3β, interacts with PI3K, and the E2-ER-GSK3β signaling loop may determine its expression^67^. Thus, dose-dependent decreases in PELP1 secretion by both cromolyn and F-cromolyn may contribute to the ameliorating anti-inflammatory activation of HMC3 microglia observed in our experiments through modulation of downstream PI3K, GSK3β, and NF-κB signaling pathways.

The heat shock protein HSP90 is a diverse chaperone protein with regulatory roles in GSK-3β stabilization and tau phosphorylation^68^ and downstream phosphorylation of Akt and PI3K/Akt/mTOR signaling^69,70^. HSP90 is also associated with the pro-inflammatory release of cytokines such as IL-6, IL-1β, and TNF-α, and the inhibition of the constitutive cytoplasmic HSP90β isoform^71^ decreases pro-inflammatory cytokine signaling, ERK phosphorylation, and STAT3 in the cytosol in N9 microglia^72^. Studies with TNFΔ^ARE^ mice with high circulating levels of TNF-α have shown that novobiocin-mediated inhibition of the C-terminal ATPase of HSP90 prevents Akt stabilization and increases its degradation, resulting in a subsequent decrease in TNF-α production^73^. Others found that HSP90β inhibition by novobiocin in the Hs578T cell line lowers soluble and insoluble fibronectin levels suggesting that, in addition to HSP90β binding directly to fibronectin, HSP90β is also involved in ECM assembly^74^. Interestingly, affinity chromatography was previously utilized to find that cromolyn binds to the N-terminus, but not to the C-terminus, of wildtype HSP90 to inhibit its N-terminal chaperone capabilities^75^. In our experiments, treatment with cromolyn and F-cromolyn results in a significant decrease in HSP90 after the addition of TNF-α. Thus, the modulation of HSP90 secretion by HMC3 microglia may be partly responsible for the upstream anti-inflammatory capabilities of cromolyn and F-cromolyn, in addition to downstream PI3K/Akt/mTOR and neurodegenerative GSK-3β signaling.

Collagens are the main structural proteins of the extracellular matrix, and there are many isoforms located within the body. Expression of collagen IV, a major component of the basal laminae, and fibronectin was found to be increased in frontal and temporal cortex cerebral microvessels in early Alzheimer’s Disease and was positively correlated with amyloid deposition compared to controls^26^. Collagen XVIII associates with vascular deposits of Aβ and senile plaques, but not tau, and is deposited at higher rates in the AD brain than in age-matched controls^76^. Interestingly, the C-terminal fragment of collagen XVIII is endostatin, an anti-angiogenic protein^77^. Endostatin has strong associations with neurological disease and can dose-dependently bind and sequester nerve growth factor (NGF) to prevent neurite outgrowth and migration in PC12 cells^77^. Increased collagen XVIII secretion has also been associated with increased VEGF expression in experiments with hepatocellular carcinoma^78^. We also observe increased concomitant secretion of collagen XVIII and VEGF (Figs. 2c, 3c, respectively), but also subsequent decreases in both proteins after cromolyn and F-cromolyn treatment.

Collagen production and fibrosis progression are intimately linked, and PI3K/Akt signaling has been associated with both^79–81^. COL6A3 is implicated as the main isoform of collagen VI that promotes downstream pro-fibrotic regulation of myofibroblasts^82^. COL6A3 depletion results in reduced migratory ability of myofibroblasts and reduced recruitment of THP-1 myeloid cells to myofibroblast-conditioned media^82^. We observed significant downregulation of COL6A3 gene expression by TNF-α induced HMC3 microglia by 30 µM cromolyn and 30 µM F-cromolyn (− 2.46 and − 2.03 fold change from TNF-α treatment alone, respectively) (Supplementary Figures S2a and S3a). Additionally, gene expression of other collagens significantly decreased by cromolyn after TNF-α administration include COL6A3, COL1A2, COL9A3, and COL8A1 (Supplementary Figure S2a). F-cromolyn also significantly reduced gene expression of COL15A1, COL6A3, COL7A1, COL9A3, COL16A1, and COL8A1 relative to treatment with TNF-α alone (Supplementary Figure S3a). Chronic immobilization stress of rats found extensive remodeling of the hippocampus with increased ECM production and reduced ECM degradation, linking the increased capacity of collagen synthesis with chronic inflammation and immunological dysregulation of the hippocampus^83^. Thus, downregulation of collagen gene expression and secretion by cromolyn and F-cromolyn in HMC3 microglia in the presence of pro-inflammatory TNF-α may affect Akt/PI3K signaling and may attenuate microglia-mediated fibrotic mechanisms initiated during chronic neuroinflammation.

Fibronectin is another essential glycoprotein component of the extracellular matrix in all body tissues, and its deposition is upregulated during fibrosis^84^. Fibronectin functions in a regulatory role for healthy deposition of ECM for use as a structural scaffold, in addition to modulating cell function during tissue repair^84^. This function continues into the CNS, as fibronectin binds collagens, proteoglycans, and other ECM proteins to reinforce the endothelial basement membrane of the BBB^85^. As seen with multiple sclerosis (MS), aberrant amounts of aggregate fibronectin deposition lead to abnormal ECM structure and impaired remyelination^86^. Normally these aggregates activate cellular repair mechanisms that then activate microglia and macrophages to a phagocytic phenotype. Still, in the presence of IFN-γ, aggregate fibronectin was shown to perpetuate inflammation, particularly by increased macrophage and microglia integrin-dependent production of nitric oxides^86^. Interestingly, cromolyn has been previously shown to inhibit mast cell-derived histamine to decrease hepatic fibrosis in Mdr2^-/-^ mice^87^. Administering cromolyn reduces hepatic fibrosis in Mdr2^-/-^ mice through inhibition of mast cell-derived histamine^87^. Mdr2^-/-^ mice have significantly higher expression of fibronectin, collagen type-1a, and α-SMA than wildtype mice, and cromolyn administration significantly reduced expression of all three^87^. Further experiments were pursued with cholestatic rats, and cromolyn decreased expression of collagen type-1a, fibronectin, and α-SMA in cultured HSCs treated with mast cell supernatants from BDL rats^88^. We observe in our study that TNF-α induced HMC3 microglia secretion of fibronectin was significantly abated by F-cromolyn treatment, suggesting that inflammation-induced fibronectin expression and potential abnormalities in ECM structure may be stemmed upstream by F-cromolyn.

Epithelial cytoskeletal proteins such as actin and keratin allow for directed integrin signaling that results in fibronectin ECM assembly^89^, with integrins serving as nucleation points for the cytoskeleton of epithelial cells and ECM proteins of the basement membrane^90^. Intriguingly, keratins 5 and 8 are known to be expressed in the choroid plexus, the region of the brain that produces CSF, forms the CSF-blood barrier, and is the only region of the brain with significant amounts of epithelial cells^91^. Keratin-1 (KRT1) is found to be excreted in extracellular vesicles by BV2 microglia^92^. It remains unclear what role these keratins play in the CNS or neurodegenerative disease, but keratin-9 (KRT9) has been proposed to be a diagnostic biomarker of AD as it has been found exclusively in AD patients’ CSF compared to controls^93^. In our experiments, it is interesting that control HMC3 microglia passively secrete KRT1, KRT5, and KRT9 and that keratin secretion is not significantly affected by TNF-α-induced inflammation (Fig. 2c). We observe that only 3 μM cromolyn treatment significantly decreases secretion of each keratin below their respective control levels (Fig. 2c), but further studies will be necessary to more fully understand the impact secreted keratin proteins have within the CNS.

Tenascin-c (TNC) is an extracellular matrix glycoprotein that promotes tissue fibrosis in many disease models, including liver, lung, MS, and systemic sclerosis, and has strong associations with other inflammatory diseases, including cancer, asthma, and Alzheimer’s disease^94^. TNC is readily expressed after injury or during inflammation and is important for efficient tissue repair but has also been associated with pathological Aβ plaque deposition with concomitant reactive glia^95^. TNC induces HDAC1 expression to promote IL-6 and TNF-α secretion by microglia; these effects were strongly associated with TLR4 signaling, with additional effects on microglia phagocytosis and migration^96^. TNC activation of TLR4 also promotes collagen synthesis and fibroblast activation and is strongly associated with the pathogenesis of systemic sclerosis in mice^33^. In our experiments, TNC secretion was modestly reduced by cromolyn and significantly decreased by F-cromolyn treatment after TNF-α administration to HMC3 microglia. In the context of microglial activation, others have shown that pretreatment of cromolyn before induced degranulation of brain mast cells reduced TLR4 signaling by microglia and subsequent inhibition of MAPK and Akt inflammatory pathways in Sprague Dawley rats^97^. As TNF-α can induce expression of TNC in HepG2 hepatoma cells^98^, the reduction of TNF-α-induced TNC secretion by F-cromolyn may also reduce microglia TLR4 and Akt signaling to prevent downstream inflammatory cytokine production and fibrosis-related signaling.

Tyrosination of microtubules is essential for normal brain development and is readily found in the dendrites and growth cones of neurons^99,100^. α-tubulin in microtubules undergoes a tyrosine removal and addition cycle, the latter of which is catalyzed by tubulin-tyrosine ligase (TTL)^101^. Studies with TTL-null mice found that TTL was essential to maintain timely neurite outgrowth and prevent early axon differentiation, emphasizing that TTL is important for the formal structure and differentiation of microtubules in neurons^101^. This stabilization during microtubule development by TTL may also be important for axon repair post-injury, as TTL regulation of tyrosinated α-tubulin has been associated with maintenance of injury signals necessary to activate axon regeneration^102^. Cromolyn and F-cromolyn both induce secretion of TTL by TNF-α activated microglia in our experiments, a factor that may contribute to the stabilization of axonal repair mechanisms during injury and subsequent regeneration of neurites.

Cold shock domain-containing E1 (CSDE1) is an RNA-binding protein that can directly interrupt transcription and translation of proteins and has been shown to prevent neurogenesis in human embryonic stem cells and iPSCs, with dozens of known neurogenesis-related transcripts significantly altered^103^. Gene variants of CSDE1 with a heightened number of binding targets, particularly with FMRP, have been associated with autism spectrum disorder^104^. Interestingly, knockdown of CSDE1 in primary mouse cortical neurons resulted in increased neurite and axon length but with fewer and thinner dendritic spines compared to controls, suggesting that CSDE1 is important for the successful morphological outgrowth of neurons^104^. CSDE1 has also been found essential to the formation of stress granules in HeLa cells^105^. Stress granules form within many cell types during extended periods of cellular stress, including oxidative stress, heat shock, TNF-α exposure, and aging, and have been postulated to function as scaffolds for aggregation-prone proteins in Alzheimer’s disease and other neurodegenerative disorders^106,107^. Our results confirm that TNF-α administration strongly induces CSDE1 secretion in HMC3 microglia (Fig. 4d). We interpret the significant decreases in CSDE1 secretion following cromolyn and F-cromolyn treatment as having the potential to reduce adverse cellular stress responses that may lead to downstream neurodegeneration and promote neurite outgrowth in coordination with NGF as observed in our PC12 cellular experiments.

Prospero homeobox protein 1 (PROX1) activates NFAT signaling pathway to promote IL-2 transcription in T cells and is associated with terminal differentiation of neural progenitor cells (NPCs) into mature neurons by inhibiting Notch 1 signaling^108^. This pathway is associated with increases in inflammatory gene expression via NF-κB, thereby increasing expression of pro-inflammatory cytokines IL-1β, TNF-α, and toll-like receptor agonists^109^. Interestingly, oligodendrocyte progenitor NG2+ cells proliferation and differentiation is PROX1-dependent, with PROX1 playing a protective role against destructive glia response of macrophages by upregulating NG2+ cells during the regenerative response to demyelination^110^. PROX1 also plays an important role in suppressing Ca^2+^ signaling and subsequent neurite outgrowth in mouse and human neuroblastoma cell lines, suggesting that transient PROX1 expression allows newly-formed neurons time to migrate into place before the extension of neurites and axons^111^. Although cromolyn and F-cromolyn significantly increase the expression of PROX1 in TNF-α induced HMC3 microglia, we do not observe deleterious effects by cromolyn or F-cromolyn in terms of neurite length of PC12 neuronal cells in our experiments. It is possible that cromolyn and F-cromolyn may reduce inflammation by increasing microglial expression of PROX1 to enhance NG2+ cell proliferation in vivo to help curb microglia-related neurodegeneration^110^, but further experiments will be required to confirm if this is an operational mechanism of cromolyn and F-cromolyn.

Ras-related protein 35 (Rab35) is a GTPase activated upstream of PI3K-Akt signaling^112^ that regulates neurite outgrowth in response to NGF by recruiting and complexing with MICAL-L1 and centaurin-β2/ACAP2 to form a site on Arf6+ endosomes for EHD1 association^113^. It was later found that Rab35 serves as a master regulator of other downstream Rab proteins to bind with MICAL-L1 to modulate neurite outgrowth after NGF administration to PC12 cells^114^. Experiments with neuronal CAD cells show Rab35 is necessary to form tunneling nanotubes^115^—protruding F-actin and tubulin cellular structures involved in vesicle trafficking and cell-to-cell communication—and macrophage inflammatory activation by LPS/IFN-γ can impair their formation^116^. Thus, Rab35 plays multiple roles in vesicle trafficking that modulate the function and growth of neuronal protrusions, and cromolyn significantly increases Rab35 secretion by TNF-α-induced HMC3 microglia. In conjunction with our neurite outgrowth experiments with PC12 cells, the increased neurite length observed with cromolyn treatment beyond NGF treatment alone may be related to cromolyn’s promotion of Rab35 upstream of PI3K/Akt signaling.

**Figure 6:**
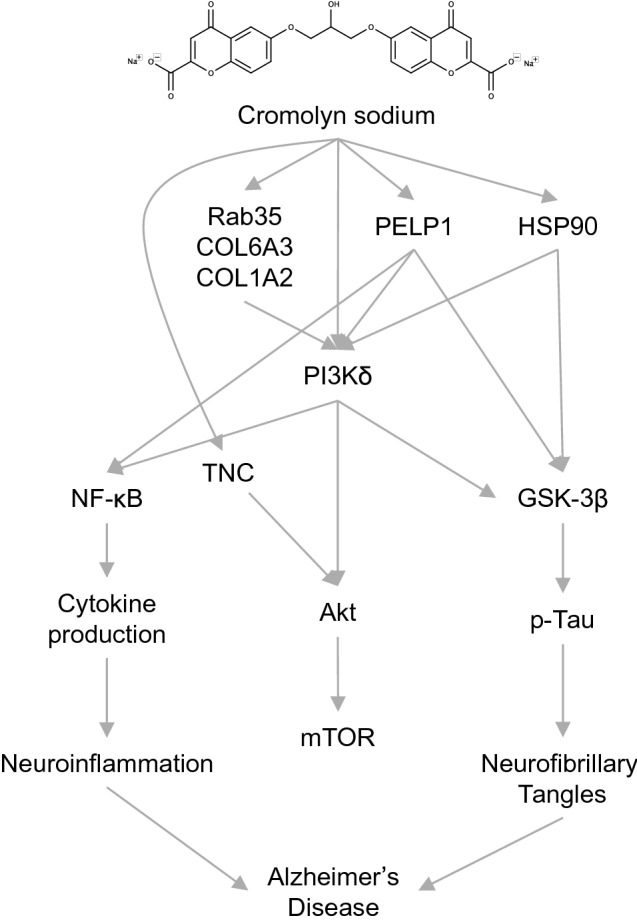
Hypothesis for the upstream modification by cromolyn and F-cromolyn to significantly affect many factors along the way to Alzheimer’s disease.

Encouraging the phagocytic potential of microglia is crucial to maintaining anti-inflammatory clearance of damage-associated molecular patterns, such as cellular debris of dead or dying cells, misfolded or aggregated proteins, and reduction of cell surface marker expression that signal inflammation from the local and peripheral environment^117^. In Alzheimer’s disease, microglia colocalize with amyloid plaques primarily consisting of Aβ, a key activator of the inflammatory microglia phenotype, that is recognized by microglia via Fc receptors, toll-like receptors, CD36, RAGE, and scavenger receptors^118^. Amyloid plaques are known neurotoxic entities of AD. Aβ is associated with increased reactive oxygen species (ROS) production^119^, and its aggregate states pair with synaptotoxicity and neuronal loss of function with underlying inflammation^120^. Thus, the ability of CNS microglia to identify and remove aggregated Aβ is a necessary component of an effective treatment strategy for AD. Cromolyn has been previously shown to upregulate the phagocytosis of Aβ42 in BV2 microglial cells^39^. We found in the present study that the ability of cromolyn and F-cromolyn to promote iPSC-derived microglia to phagocytize Aβ42 is upregulated relative to controls, further indicating that the cromolyn platform can influence microglia towards an anti-inflammatory phenotype. Figure 6 summarizes the proposed waypoints along the path to Alzheimer’s disease that cromolyn and F-cromolyn can measurably affect.

## Conclusion

Neuroinflammation is a significant component of most neurological disorders—including ALS, MS, AD, Huntington’s, and others. Chronic inflammation leads to increased neuronal damage that provides a positive feedback mechanism that prolongs inflammatory microglial activation. Prolonged inflammation leads to the recruitment of leukocytes and other peripheral cells to damaged tissue instead of maintaining microglial clearance mechanisms. In the context of chronic inflammation, the body’s wound healing process becomes damaging to brain function. Excessive ECM deposition and glial scarring can obstruct neurons and stress their processes, inhibiting their ability to function normally. There is currently no approved treatment regimen available to tackle the multi-factorial nature of neurodegenerative disorders to slow or prevent disease. An efficacious therapeutic will be required to address disease-related triggers of plaque and tau tangle formation, the neuroinflammatory response, fibrosis, and the promotion of neurogenesis. Here we present evidence that cromolyn and F-cromolyn can dampen inflammation in HMC3 human microglia induced by TNF-α and promote the secretion of anti-inflammatory IL-4. We also show that cromolyn and F-cromolyn reduce key components of the ECM, including fibronectin and tenascin-c, that have been related to fibrosis. Additionally, we verify that cromolyn and F-cromolyn augment NGF’s capability to encourage neurite outgrowth of PC12 cells and promote phagocytosis of neurotoxic Aβ42 by iPSC-derived microglia; which cromolyn may accomplish through targeting PI3K signaling. Altogether, the cromolyn drug platform represents a robust treatment strategy for multiple aspects of neurodegenerative diseases that currently have no approved therapeutic.

## Supplementary Information

Supplementary Figures.
