# Supplementary material for: Cromolyn platform suppresses fibrosis and inflammation, promotes microglial phagocytosis and neurite outgrowth
Source: Sci Rep. 2021 Nov 12;11:22161. doi: 10.1038/s41598-021-00465-6 (PMC8589953; doi:10.1038/s41598-021-00465-6)
Supplement: Supplementary file 1 — Supplementary Figures. [file 41598_2021_465_MOESM1_ESM.docx]

**Supplementary Materials for:**

**Cromolyn Platform Suppresses Fibrosis and Inflammation, Promotes Microglial Phagocytosis and Neurite Outgrowth**

**Yi-Jun Wang^1^, Matthew A. Downey^1^, Sungwoon Choi^2^, Timothy M. Shoup^3^ and David R. Elmaleh^1,3,*^**

Affiliations: ^1^AZTherapies, Inc., Boston, MA, USA; ^2^Department of New Drug Discovery, Chungnam National University, South Korea; ^3^Department of Radiology, Massachusetts General Hospital and Harvard Medical School, Boston, MA, 02129-2060, USA

*Correspondence to: David R. Elmaleh, PhD, Massachusetts General Hospital, Department of Radiology, 55 Fruit Street, Boston, MA 02114, USA. Tel.: +1 617 318 3430; Fax: +1 617 848 8703; E-mail: delmaleh@mgh.harvard.edu.; E-mail: delmaleh@aztherapies.com.

**Supplementary Figures**

 **Supplementary Figure S1**. TNF-α potently induces differential expression of a wide array of fibrosis and inflammation-associated genes and proteins. **(a)** Table summarizes the top 20 Up-regulated and Down-regulated genes upon addition of 0.3 µg/mL TNF-α to HMC3 microglia, which is derived from the volcano plot dataset. Association of these changed genes were annotated by the color of asterisk: yellow for neuroinflammation, red for inflammation, and blue for fibrosis. **(b)** Enriched pathway network plot shows that TNF-α significantly alters many cellular pathways that inter-relate with fibrosis in HMC3 microglia, including interconnections of ECM organization, collagen biosynthesis/assembly, collagen formation, and ECM degradation. **(c)** Table summarizes the significantly enriched biological process GO terms that is derived from the String-db functional analysis of Protein-Protein Interaction Network (PPINet). GO terms were annotated by color: red for response to cytokines, blue for cytokine-mediated signaling pathway, green for response to stress, and yellow for chemokine-mediated signaling pathway.

 **Supplementary Figure S2.** Cromolyn reduces expression of fibrosis and inflammation-associated genes and proteins induced by TNF-α in HMC3 human microglia. **(a)** Table summarizes the top 20 Up-regulated and Down-regulated genes upon 30 µM cromolyn addition to 0.3 µg/mL TNF-α induced HMC3 microglia as compared to TNF-α alone, which is derived from the volcano plot dataset. Association of these changed genes were annotated by the color of asterisk: yellow for neuroinflammation, red for inflammation, and blue for fibrosis. **(b)** Enriched pathway network plot shows that 30 µM cromolyn addition to TNF-α treated HMC3 microglia significantly alters many cellular pathways that inter-relate with fibrosis in HMC3 microglia as compared to TNF-α alone, including interconnections of ECM organization, degradation of ECM, assembly of collagen fibrils and collagen degradation.

 **Supplementary Figure S3**. F-cromolyn reduces expression of fibrosis- and inflammation-associated genes and proteins induced by TNF-α in HMC3 human microglia. **(a)** Table summarizes the top 20 Up-regulated and Down-regulated genes upon 30 µM F-cromolyn addition to 0.3 µg/mL TNF-α induced HMC3 microglia as compared to TNF-α alone, which is derived from the volcano plot dataset. Association of these changed genes were annotated by the color of asterisk: yellow for neuroinflammation, red for inflammation, and blue for fibrosis. **(b)** Enriched pathway network plot shows that 30 µM F-cromolyn addition to TNF-α treated HMC3 microglia significantly alters many cellular pathways that inter-relate with fibrosis in HMC3 microglia as compared to TNF-α alone, including interconnections of collagen fibril assembly and neutrophil degranulation. **(c)** Cytokine secretion of HMC3 microglia controls (white diamonds), after addition of 0.3 µg/mL TNF-α (yellow squares), 0.3 µg/mL TNF-α + cromolyn (0.3μM, 3μM, 10μM, 30μM, blue circles), and 0.3 µg/mL TNF-α + F-cromolyn (0.3μM, 3μM, 10μM, 30μM, green triangles) for GRO-α. ***p < 0.001, **p < 0.01, *p < 0.05.

 **Supplementary Figure S4**. Neurite outgrowth and neurogenesis in PC12 cells. **(a)** Representative fluorescent micrographs show nerve growth factor (NGF) significantly promote neurite outgrowth in PC12 neuronal cells in a concentration-dependent manner. PC12 cells were stained with β3-tubulin (green) for neurite outgrowth, and with Hoechst (blue) for nuclei. **(b)** Representative fluorescent micrographs show that NGF significantly promote neurite outgrowth in PC12 cells at 100 ng/ml.

**Supplementary Figure S5**. iPSC-derived human microglial phagocytosis of amyloid β-protein 42 (Aβ42). **(a)** Representative fluorescent micrograph of IBA-1 staining shows that these iPSC-differentiated microglial cells have high microglial purity of 95%. **(b)** Quantitative analyses of microglial phagocytosis suggest that the microglial cell number of 2,000 cell/well and the FITC-labeled Aβ42 concentration of 0.3µM are the optimized conditions to conduct compound evaluation. **(c)** Representative fluorescent micrographs show 1 µM of FITC- Aβ42 significantly promoted microglial phagocytosis. FITC-Aβ42 (green), lysotracker for cell lysosome (orange), and Hoechst nuclei stain (blue).
